# The cost of post-abortion care (PAC): a systematic review

**DOI:** 10.1186/s12913-022-07765-1

**Published:** 2022-03-25

**Authors:** Estro Dariatno Sihaloho, Ibnu Habibie, Fariza Zahra Kamilah, Yodi Christiani

**Affiliations:** 1 Departemen of Economics, Universitas Padjadjaran, Sumedang, Indonesia; 2 Yayasan Inisitatif Perubahan Akses Menuju Sehat (IPAS) Indonesia, Jakarta, Indonesia

**Keywords:** Cost of post-abortion care (PAC), Medical vacuum aspiration (MVA), Dilatation and Curettage (D&C)

## Abstract

**Background:**

Despite the increasing trend of Postabortion Care (PAC) needs and provision, the evidence related to its cost is lacking. This study aims to review the costs of Postabortion Care (PAC) per patient at a national level.

**Methods:**

A systematic review of literature related to PAC cost published in 1994 – October 2020 was performed. Electronic databases such as PubMed, Medline, The Cochrane Library, CINAHL, and PsycINFO were used to search the literature. Following the title and abstract screening, reporting quality was appraised using the Consolidates Health Economic Evaluation (CHEERS) checklist. PAC costs were extrapolated into US dollars ($US) and international dollars ($I), both in 2019.

**Results:**

Twelve studies met the inclusion criteria. All studies reported direct medical cost per patient in accessing PAC, but only three of them included indirect medical cost. All studies reported either average or range of cost. In terms of range, the highest direct cost of PAC with MVA (Medical Vacuum Aspiration) services can be found in Colombia, between $US50.58–212.47, while the lowest is in Malawi ($US15.2–139.19). The highest direct cost of PAC with D&C (Dilatation and Curettage), services is in El Salvador ($US65.22–240.75), while the lowest is in Bangladesh ($US15.71–103.85). Among two studies providing average indirect cost data, Uganda with $US105.04 has the highest average indirect medical cost, while Rwanda with $US51.44 has the lowest.

**Conclusions:**

Our review shows variability in the cost of PAC across countries. This study depicts a clearer picture of how costly it is for women to access PAC services, although it is still seemingly underestimated. When a study compared the use of UE (Uterine Evacuation) method between MVA and D&C, it is confirmed that MVA treatments tend to have lower costs and potentially reduce a significant cost. Therefore, by looking at both clinical and economic perspectives, improving and strengthening the quality and accessibility of PAC with MVA is a priority.

**Supplementary Information:**

The online version contains supplementary material available at 10.1186/s12913-022-07765-1.

## Background

Despite a 35 percent decline over a seven-year period, maternal deaths are still considered high in 2017, with an estimated 295,000 deaths worldwide [1]. While the causes are varied, the WHO predicted unsafe abortion had contributed to 13% of all maternal deaths [2]. Unsafe abortion is defined as the termination of an unwanted pregnancy procedure that is performed by unqualified persons, or in an environment with minimal medical standards, or both [3, 4]. Between 2010 and 2014, it is projected that 25.1 million cases of unsafe abortion occurred worldwide, with 97 percent of them occurring in developing countries [5]. However, due to highly restrictive laws, providing abortion services that are safe for women remains a challenge [6]. For example, in 68 countries abortion is strictly prohibited and only permitted to save a woman’s life [7].

Under restrictive legal settings, many women with an unwanted pregnancy tend to seek unsafe abortions and they will potentially experience severe health complications as a result [8]. To manage complications, they need treatment, namely Postabortion Care (PAC) [9]. The Guttmacher Institute projected that more than 20 million women in Low-Middle Income Countries (LMICs) with unsafe abortion need PAC treatment. Still, only about 60% of them received it [10], mostly because of low-quality health systems [11].

Providing PAC treatment is a financial challenge for a government, especially because the severity of complications experienced by women varies. To illustrate, in Sub-Saharan Africa, Vlassoff et al. [12] indicated the annual health-care costs of treating unsafe abortion complications are projected to be between $68 million and $76 million. The cost also depends on the methods used in PAC treatment which commonly include Curettage and Dilatation (D&C), Manual Vacuum Aspiration (MVA), and medicamentosa methods.

The aim of this study was to explore the variation of cost of accessing PAC per patient at a national level across countries. We conducted a systematic review and extrapolated the cost into the international dollar for comparing purposes, where appropriate. Further, a comparison of costs can be used as a reference for the government in setting rules regarding the PAC method to be used nationally and strengthening and providing high-quality PAC services.

## Methods

### Search strategy

The review was guided by The PRISMA statement [13] and the CHEERS statement [14]. A systematic review was performed using electronic databases including, PubMed, Medline, The Cochrane Library, CINAHL, and PsycINFO. Inclusion criteria include, available manuscripts from aforementioned databases and hand searching from reference lists in the potential selected articles published from January 1, 1994 (the year the PAC initiative was introduced [15]) to October 10, 2020. Other inclusion criteria are title, publications only in the English language, and the study should examine at least direct medical cost (i.e., costs of drugs and supplies and costs of personnel). We did not use geography as an inclusion. We excluded grey literature, such as institutional and donor reports, and studies that were not original articles and have been superseded were also excluded.

We developed the search term for health-system-cost related from Gordon and Rowell [16] and for postabortion care related terms from Tripney, et al. [15] as described in Appendix 1. Before conducting the review, we developed a protocol as the guideline. Two researchers performed the searches and conducted literature screening individually to select potential studies. When discrepancies occurred regarding the studies’ inclusion, both researchers discussed reaching a consensus.

In this paper, we focus on the costs incurred by health facilities, namely direct medical costs and indirect medical costs. Direct medical costs consist of labor costs and supplies costs while indirect medical costs consist of overhead costs and capital costs.

### Study quality

Research quality appraisal was guided by CHEERS statement – a specific instrument or guidelines developed for studies reporting economic outcomes as they tend to require additional economic data such as resource use, costs, and effectiveness results [14]. Hence, this instrument enabled us to critically appraise study findings, extract components of PAC cost, and assess the eligibility for being included in the extrapolated cost. However, we chose items related to our cost analysis study only. The critical appraisal was conducted by two independent reviewer authors, and the results were discussed with the remaining author. Discrepancies were discussed and a consensus was reached during the discussion. As a result, all authors agreed to include studies that fulfill any criteria below:

1. Fully reported direct medical costs, personnel costs, supplies costs, indirect medical costs, overhead costs, and capital costs.
2. Only reported direct medical costs and indirect medical costs
3. Only reported personnel costs, supplies costs, overhead costs, and capital costs

### Field and study setting

Twelve studies were located in Eastern Africa (Ethiopia [17]; Malawi [18]; Rwanda [19]; Uganda [20]; Tanzania [21]), Western Africa (Burkina Faso [22]; Nigeria [23]; Senegal [24]; Sierra Leone [25]), Southern Asia (Bangladesh [26]), South America (Colombia [27]), and Central America (El Salvador [28]). All of these countries are in a group of Low-Middle Income Countries (LMICs) based on World Bank classification, with seven of them being low-income countries.

### Data extraction, synthesis, and cost extrapolation

Data was extracted for each eligible study by two reviewer authors independently. Data extracted includes published year, time of data collection, cost estimation method, and estimated direct medical cost, labour or personnel cost, supplies and drugs or medications cost, indirect medical cost, overhead cost, and capital cost. No discrepancies were found in the data extracted by the two authors. We followed several studies for developing operational definitions, as shown in Table 1.

**Table 1:** Operational definitions of costs by category

Name	Components	Explanations/sources
Direct Medical Cost	Labor Cost	The costs of time spent treating a patient by all medical personnel involved in a patient’s care [19, 24]
	Supplies and Drugs Cost	Small equipment, diagnostic tests, medications, and consumable supplies [24, 28]
Indirect Medical Cost	Overhead Cost	Salaries of non-medical support staff (e.g., receptionists) and administrative costs incurred by clinical staff (e.g., attending meetings) [19, 24, 28]
	Capital Cost	Infrastructure and equipment replacement costs and their useful lifetimes [19]

A deductive approach content analysis was used to summarize the data. PAC costs from each study were converted into two forms, US dollars ($US) and international dollars ($I), both in 2019, to improve the comparability and transferability of results across studies. An international dollar is a hypothetical unit of currency used for comparisons between countries, and at a given point in time, it has the equivalent purchasing power parity of the US dollar in the United States [12].

The cost extrapolation process starts with adjusting costs in US dollars ($US) in the year of the study for inflation using the US GDP deflator to obtain the PAC cost in US dollars ($US) in 2019. Afterward, costs are converted into the country’s local currency unit from each study in 2019 using the official exchange rate, then divided by the PPP of each study country to obtain the PAC cost in international dollars ($I) in 2019. This PPP is used to equalize purchasing power between countries by eliminating differences in price levels [29], which makes international comparison possible. All data used to convert PAC costs, such as GDP deflator, exchange rates, and PPP, are obtained from the World Development Indicator by the World Bank [30].

## Results

**Fig. 1:**
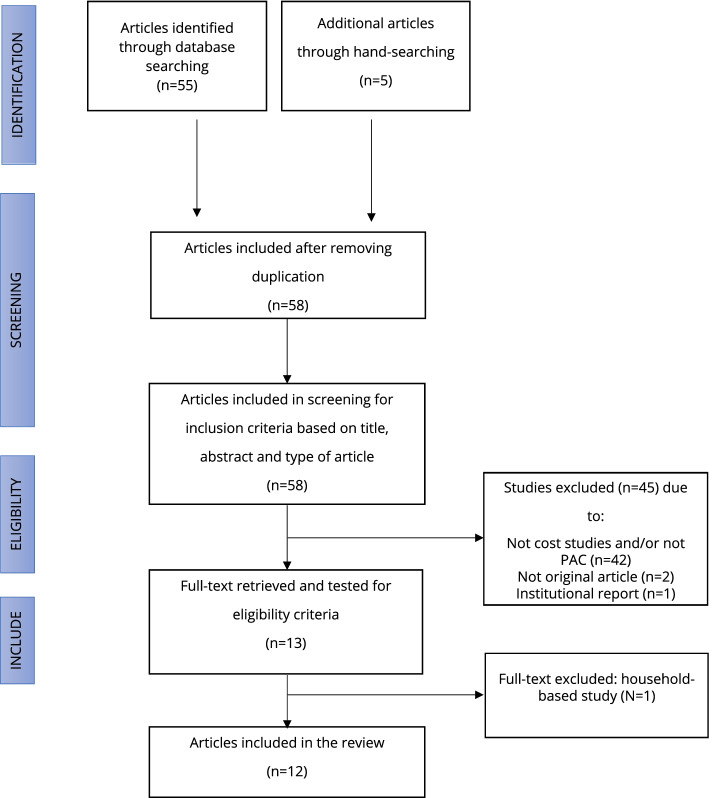
PRISMA diagram

Based on the first search, we found 275 potentially relevant papers throughout the sources. After review based on titles as well as excluded duplication, a total of 58 distinct articles were selected for initial screening based on titles and abstracts (Fig. 1). From this step, we dropped 45 articles out of 58 articles, of which 42 articles were not related to a cost analysis study or did not specifically cover the issues of PAC. The other two studies were not original articles, and the third was an institutional report.

A total of 10 full-text articles were retrieved to assess for more detailed evaluation, including examining their quality and clarity in terms of reporting using the CHEERS statement. From this process, three studies were excluded because one study was based on a household perspective and other chosen studies superseded the remaining two studies. We then manually searched potential articles from the reference lists of the seven articles and found five studies to include. Table 2 summarizes information related to the twelve chosen studies, including their reported component cost.

**Table 2:** Summarize information from 12 chosen studies ^+−^

Authors	Published year	Country	Timing	Costing tools	Base Currency ($USD)	Component cost					
						**Direct Medical Cost**	**Labor Cost**	**Supplies and Drugs Cost**	**Indirect Medical Cost**	**Overhead Cost**	**Capital Cost**
Benson J, et al. [18]	2015	Malawi	May to June, 2010	savings, excel-based	2010	+	-	-	-	-	-
Ilboudo, et al. [22]	2016	Ouagadougou, Burkina Faso	April 2010 to 2011	Excel spreadsheet	2010	+	+	+	-	+	-
Benson J, et al. [23]	2012	Nigeria	June to September, 2010	savings, excel-based	2012	+	-	-	-	-	-
Johnston HB, et al. [26]	2012	Bangladesh	June to August, 2008	Excel spreadsheet	2008	+	+	+	-	-	-
Prada E, et al. [27]	2013	Colombia	January to April, 2012	Post-Abortion Care Costing sMethodology (PACCM)	2012	+	+	+	-	-	-
Vlassoff M, et al. [17]	2012	Ethiopia	2008	Post-Abortion Care Costing Methodology (PACCM)	2008	+	-	-	-	-	-
Lince-Deroche N, et al. [24]	2020	Senegal	September 2016 to January 2017	Post-Abortion Care Costing Methodology (PACCM)	2016	+	+	+	+	+	+
Baynes C, et al. [21]	2019	Tanzania	June to September, 2017	Excel spreadsheet	2016	-	+	+	-	+	+
Koontz SL, et al. [28]	2003	El Salvador	February to November, 1999	Modified Ipas cost model	2003	+	+	+	-	-	-
Vlassoff M, et al. [19]	2015	Rwanda	April to May, 2012	Post-Abortion Care Costing Methodology (PACCM)	2012	+	+	+	+	+	+
Vlassoff M, et al. [20]	2014	Uganda	August to November, 2010	Post-Abortion Care Costing Methodology (PACCM)	2010	+	+	+	+	+	+
Paul M, et al. [25]	2015	Sierra Leone	Jun to July, 2012	excel-based, Modified Delphi approach	2012	+	-	-	-	-	-

Table 1 shows that the component cost reported were varied across the studies

^+^ Means Available in the Studies

^−^ Means Not Available in the Studies

Table 2 shows that the component costs reported were varied across the studies. The variability is caused by several factors, such as available data and chosen tools. However, all studies reported the direct medical cost of postabortion care per patient at a national level. It should be noted, regardless of not reporting the direct medical cost explicitly, we decided to include a study in Tanzania [21] because they reported labour cost and supplies and drug cost, which are components for direct medical cost based on our operational definition. Also, the variability occurred in terms of reporting the cost of each component. While some only reported their average value [17, 19, 20], the others provided the cost by severity, resulting in a range of cost when summarized in this study. Those who did not report the average values tend to avoid any misleading results because the nature of PAC services is not typically generalizable, as a small number of cases can be quite disproportionate to common cases. This is due to the different levels of severity in PAC cases. Some studies determine the cost range from the simplest complications to the most severe complications and decide not to report the average values.

In terms of approaches, costing analysis can be divided into two types, a “top-down” and a “bottom-up”. When a study uses the first approach, it scrutinizes large administrative datasets and derives PAC-related cost for estimating its overall cost per patient [12, 16]. When it uses the latter approach, at first, it identifies and estimates each component then adds it up for the final estimation. All chosen studies tend to use a bottom-up approach, meaning they first collect data on component costs (i.e., direct costs and indirect medical costs) then compose them into the cost of PAC. However, some of them also used a large number of the patient database to estimate resources used, such as a study in El Salvador, which emphasized that their methodology is more specific to patient-derived costs [28].

Most of the studies used the PACCM as their tools. The PACCM is a bottom-up ‘ingredients’ approach that relies on expert opinion for estimating the health-system-cost component [20]. A study in Sierra Leone used the modified Delphi approach to solicit information [25]. Two studies in Nigeria and Malawi used *savings*, an excel-based tool to estimate per-case PAC costs [18, 23].

Table 3 describes (i) direct medical costs, (ii) labour costs, and (iii) supplies and drugs costs, in 2019 US dollars and international dollars, of PAC from 12 countries. From the table above, the costs show a lot of variabilities. There are three types of costs reported in the table, namely cost by MVA method, cost by D&C method, and unspecified abortion method costs. In terms of range, the highest direct cost of PAC with MVA services can be found in Colombia, between $US50.58–212.47, while the lowest is in Malawi ($US15.2–139.19). The highest direct cost of PAC with D&C services is in El Salvador ($US65.22–240.75), while the lowest is in Bangladesh ($US15.71–103.85).

**Table 3:** Average/range cost of direct medical cost for PAC treatment per patient

**Authors**		**($US in 2019)**			**($I in 2019)**		
		**MVA**	**D&C**	**Method of abortion were not specified**	**MVA**	**D&C**	**Method of abortion were not specified**
Benson J, et al. [18]	Direct Medical Cost	15.2—139.19	22.22—161.42	NA	40.75—373.08	59.56—432.64	NA
	Labour Cost	NA	NA	NA	NA	NA	NA
	Supplies and Drugs Cost	NA	NA	NA	NA	NA	NA
Ilboudo, et al. [22]	Direct Medical Cost	27.16—30.89	NA	NA	79.93—90.91	NA	NA
	Labour Cost	1.23—1.6	NA	NA	3.64—4.71	NA	NA
	Supplies and Drugs Cost	25.56—29.65	NA	NA	75.25—87.26	NA	NA
Benson J, et al. [23]	Direct Medical Cost	74.19—137.15	NA	NA	168.06—310.66	NA	NA
	Labour Cost	NA	NA	NA	NA	NA	NA
	Supplies and Drugs Cost	NA	NA	NA	NA	NA	NA
Johnston HB, et al. [26]	Direct Medical Cost	15.71—27.68	15.71—103.85	NA	42.13—74.23	42.13—278.46	NA
	Labour Cost	7.15—8.48	5.94—13.46	NA	19.18—22.76	15.95—36.09	NA
	Supplies and Drugs Cost	7.22—20.53	13.33—90.38	NA	19.37—55.05	35.74—242.33	NA
Prada E, et al. [27]	Direct Medical Cost	50.58—212.47	NA	NA	123.03—516.75	NA	NA
	Labour Cost	NA	NA	42.72—214.72	NA	NA	103.89—522.21
	Supplies and Drugs Cost	NA	NA	7.86—24.73	NA	NA	19.13—60.15
Vlassoff M, et al. [17]	Direct Medical Cost	NA	NA	43.17	NA	NA	120.6
	Labour Cost	NA	NA	NA	NA	NA	NA
	Supplies and Drugs Cost	NA	NA	NA	NA	NA	NA
Lince-Deroche N, et al. [24]	Direct Medical Cost	NA	NA	25.03—34.07	NA	NA	61.17—83.27
	Labour Cost	NA	NA	3.7—7.31	NA	NA	9.05—17.86
	Supplies and Drugs Cost	NA	NA	20.85—26.76	NA	NA	50.95—65.4
Baynes C, et al. [21]	Direct Medical Cost	NA	NA	NA	NA	NA	NA
	Labour Cost	NA	NA	8.94—30.06	NA	NA	21.97—73.83
	Supplies and Drugs Cost	NA	NA	11.89—25.22	NA	NA	29.21—61.95
Koontz SL, et al. [28]	Direct Medical Cost	58.82—105.39	65.22—240.75	NA	128.4—230.05	142.37—525.51	NA
	Labour Cost	13.34	16.34	NA	29.12	35.66	NA
	Supplies and Drugs Cost	11.84	15.79	NA	25.85	34.47	NA
Vlassoff M, et al. [19]	Direct Medical Cost	NA	NA	48.71	NA	NA	140.86
	Labour Cost	6.05–31,48	NA	NA	17.52–91.06	NA	NA
	Supplies and Drugs Cost	19.39–73.5	NA	NA	56.07–212.54	NA	NA
Vlassoff M, et al. [20]	Direct Medical Cost	NA	NA	41.34	NA	NA	119.86
	Labour Cost	11.55–17.8	NA	NA	33.5–51.6	NA	NA
	Supplies and Drugs Cost	25,64–48.51	NA	NA	74.32–140.65	NA	NA
Paul M, et al. [25]	Direct Medical Cost	68.57–109.04	NA	NA	243.27—386.85	NA	NA
	Labour Cost	NA	NA	NA	NA	NA	NA
	Supplies and Drugs Cost	NA	NA	NA	NA	NA	NA

Table 2 provides the summary of (i) direct medical costs, (ii) labour costs and (iii) supplies and drugs costs, in 2019 US dollars and international dollars, of psost-abortion care (PAC) from 12 countries

Table 3 also shows that only three studies reported both methods: MVA and D&C. From the three studies above (El Salvador, Bangladesh, and Malawi), the MVA cost tends to be lower than the D&C cost. In El Salvador, the cost is generally lower when MVA is used, ranging from $US58.82 to $US105.39, while D&C costs more, ranging from $US65.22 to $US240.75. In Bangladesh the cost when MVA is used is extremely lower, ranging from $US15.71 to $US27.68, while the use of D&C can cost up to $103.85. Last, in Malawi, the cost for MVA use, ranges from $US15.2 to $US139.19, while D&C costs between $US22.22 and $US161.42.

Additionally, two studies report the labor cost and supplies/drugs cost of UE using MVA & D&C, these are Bangladesh and El Salvador. For the use of MVA, the labor cost in Bangladesh ranges from $US7.15 to $US8.48 while for the use of D&C, it ranges from $US5.94 to $US13.46. For the supplies/drugs cost, the use of MVA in Bangladesh also costs less ($US7.22–20.53) compared to D&C ($US13.33—90.38). Similar to Bangladesh, the use of D&C in El Salvador generally requires more labor hours, longer stays in the hospital, and more supplies/drugs; consequently, it will raise labor costs and supplies/drugs costs.

Unlike the direct medical cost, the studies that reported the indirect cost of providing PAC did not distinguish between the use of UE method, as shown in Table 4. Among two studies that were providing average indirect cost data, Uganda has the highest average cost with $US105.04, while Rwanda has the lowest average cost with $US51.44. The high cost in Uganda was burdened by the capital cost which accounts for around 80% of the indirect medical cost, while in Rwanda that cost only accounts for less than 30%.

**Table 4:** Average/range cost of indirect medical cost for PAC treatment per patient

**Authors**		**(US$ in 2019)**			**($I in 2019)**		
		**MVA**	**D&C**	**Method of abortion were not specified**	**MVA**	**D&C**	**Method of abortion were not specified**
Ilboudo, et al. [22]	Indirect Medical Cost	NA	NA	NA	NA	NA	NA
	Overhead Cost	NA	NA	15.53	NA	NA	45.71
	Capital Cost	NA	NA	NA	NA	NA	NA
Lince-Deroche N, et al. [24]	Indirect Medical Cost	NA	NA	6.53—7.26	NA	NA	15.97—17.76
	Overhead Cost	NA	NA	2.12—2.53	NA	NA	5.18—6.19
	Capital Cost	NA	NA	0.52—0.59	NA	NA	1.27—1.45
Baynes C, et al. [21]	Indirect Medical Cost	NA	NA	NA	NA	NA	NA
	Overhead Cost	NA	NA	17.63	NA	NA	43.31
	Capital Cost	NA	NA	14.28	NA	NA	35.08
Vlassoff M, et al. [19]	Indirect Medical Cost	NA	NA	51.44	NA	NA	148.76
	Overhead Cost	NA	NA	36.17	NA	NA	104.61
	Capital Cost	NA	NA	15.25	NA	NA	44.11
Vlassoff M, et al. [20]	Indirect Medical Cost	NA	NA	105.04	NA	NA	304.49
	Overhead Cost	NA	NA	22.09	NA	NA	64.05
	Capital Cost	NA	NA	82.94	NA	NA	240.44

Table 5 shows the results of direct medical cost distinguished by five major abortion complications included in the WHO study on costing in its “Mother-Baby Package” [21]. The total amount of direct cost from complications shows a lot of variation.

**Table 5:** Average/range cost of direct medical cost, by abortion complications*

	**Average/range of direct cost by type of complication ($ in 2019)**					**Average/range of direct by type of complication ($I in 2019)**				
	**Incomplete Abortion**	**Shock**	**Sepsis**	**Lacerations**	**Perforations**	**Incomplete Abortion**	**Shock**	**Sepsis**	**Lacerations**	**Perforations**
Ilboudo, et al. [22]	24.99 -30.44	29.07—52.11	88.61—110.4	38.65—69.42	86.27	73.56—89.6	85.58—153.36	260.8—324.93	113.77—204.31	253.91
Prada E, et al. [27]	7.86—21.36	308.03—410.34	69.7—98.93	26.98—60.7	103.42—251.82	19.13—51.94	749.15—997.95	169.51—240.6	65.61—147.64	251.54—612.44
Vlassoff M, et al. [17]	NA	23.41—23.65	12.43—25.57	19.79	NA	NA	65.41—66.08	34.74—71.44	55.29	NA
Lince-Deroche N, et al. [24]	15.77	39.53	25.74	36.33	38.52	38.53	96.60	62.91	88.79	94.13
Baynes C, et al. [21]	20.43—49.17	21.52—72.9	36.78—149.84	41.94—71.32	19.99—134.29	50.19—120.77	52.85—179.05	90.33—368.01	103—175.17	49.1—329.83
Vlassoff M, et al. [19]	60.83	103.14	67.22	31.36	56.43	175.91	298.27	194.40	90.70	163.19
Vlassoff M, et al. [20]	45.96	48.63	44.48	52.76	125.61	133.25	140.99	128.95	152.95	364.13

Table 4 shows the results of direct medical cost distinguished by five major abortion complications included in the WHO study on costing in its “Mother-Baby Package” [22]

Looking from a complications’ point of view, in terms of the average amount of direct cost, Rwanda has the highest direct medical cost for incomplete abortion ($US60.83), shock ($US103.14) compared to other countries. In Senegal, the highest average cost is a result of shock ($US39.53), while its lowest is incomplete abortion ($US15.77). Lastly, in Uganda, the complication that costs the most, on average, is perforation ($US125.61), and the least is shock ($US44.48).

## Discussion

Out of the twelve papers that were included in this review, we found no study examining the indirect cost of PAC, which limits our knowledge in estimating the actual cost of PAC. Our review provides insights into the service delivery costs of providing PAC per patient at a national level across countries, particularly developing countries. As shown, the cost is varied and dependent on many factors, such as data availability, number of severe cases, period of data collection, and costing method. For example, almost half of the chosen studies used the PACCM tool, which was developed by the Guttmacher Institute and is argued as a low-cost approach yet provides a robust estimation for policy analysis [17, 19]. All studies that reported indirect medical cost used the PACCM as their tools, naming it as the most comprehensive tool for cost analysis of PAC service.

Using US dollar ($US) in 2019, the most expensive MVA case in this study is found in Colombia ($212.47), whereas the most expensive D&C case is in El Salvador ($240.75). MVA cases cost the least in Malawi ($US15.71), and D&C cases cost the least in Bangladesh ($US15.71). As illustrated in Malawi, where the cheapest PAC treatment is $US15.71 and the most expensive is $139.19, averaging the cost of PAC treatment remains difficult. Further, when comparing the cost, it first needs to be adjusted to account for the severity of the case.

In addition, to extrapolate the cost into the US dollar ($US) in 2019, this review also converted all costs into international dollars ($I). Where appropriate, this would allow us to compare the patient’s purchasing power in affording postabortion care in their respective countries to other countries. It is suggested that the use of the international dollar should be interpreted carefully as converting to the international dollar could result in differences that are plausibly caused by high rates of inflation in some countries relative to the USA [31]. For example, if we look at the international dollars, the starting highest direct medical cost of PAC services (using MVA) is no longer in Nigeria $I168.06 ($US74.19), but Sierra Leone at $I243.27 ($US68.57). This explicitly shows that, concerning patient’s purchasing power, the expensive case of PAC service can be found in Sierra Leone. Thus, one needs to ensure that the economic characteristics of one country to be compared to another (e.g., inflation rate relative to the USA) are not too dissimilar.

Some of the studies explored the cost of WHO-recommended UE methods, like MVA in more detail. Not only the cost is relatively lower than D&C, but MVA also has been clinically proven to be as effective and safer than sharp curettage for treating incomplete abortion and inducing first-trimester abortion [32]. As previously confirmed by several studies, when comparing the cost of providing UE between MVA and D&C in these countries, MVA treatments tend to have lower costs compared to D&C. The use of local anesthesia for MVA instead of general anesthesia when D&C is employed seemingly reduces the cost of MVA [26]. Also, the use of MVA potentially results in a shorter hospital stay for patients and consequently reduces labour cost, which in combination would significantly decrease the overall direct cost [26, 28].

A study in Malawi made a simulation if all women seeking first-trimester induced abortion chose MVA, the result would be an estimated 20% cost reduction [18]. A study in El Salvador inspected the use of both methods for treating incomplete abortion, which not only confirmed that MVA could safely treat incomplete abortion, with considerable decreased hospital stay and cost as compared to sharp curettage, but also revealed that MVA has a stronger link to contraceptive services and is associated with contraceptive acceptance rates [28]. Therefore, improving the quality and accessibility of PAC with MVA can reduce the overall cost of PAC and lead to alleviating the burden of unsafe abortion.

This systematic review has some limitations for consideration and discussion. First, the review only included English-language articles, which may lead to a lesser search result. Second, the differences in the operational definitions in the selected studies can potentially affect the findings of this review. In some studies [19, 20], components of labour costs were also distinct, hence it is not possible to group the costs into the same component as others. Third, the varieties in reporting the cost (range vs average). To avoid any misleading results, we did not calculate the average cost when the author did not provide it in their study. Despite its limitations, this study depicts a clearer picture of how costly it is for women to access PAC service. Still, the cost is potentially underestimated as all studies included in this review did not estimate other components such as long-term healthcare costs for any complications due to unsafe abortion.

## Conclusions

Our review shows variability in providing PAC across countries. This study depicts a clearer picture of how costly it is for women to access PAC service, although it is still seemingly underestimated. Our findings also reconfirm MVA as a method of choice for PAC. In addition to the medical safety and women-centered approach of the method, it is also shown that MVA is less costly compared to D&C. With a projected increasing demand of PAC globally, shifting UE methods to MVA will not solely benefit women but in turn, will lead to a reduced burden of cost associated with PAC across levels. Hence, shifting UE methods to MVA should be initiated and promoted. Among others, it should be started with updated policies and clinical guidelines related to it, health workers capacity buildings, and supplies at the facilities level. The government, medical professionals, and health facilities’ roles are critical in this effort.

## Supplementary Information

**Additional file 1.**

## Data Availability

The datasets used and/or analysed during the current study available from the corresponding author on reasonable request.

## Abbreviations

CHEERS: Consolidated health economic evaluation reporting standards
D&C: Dilatation and curettage
GDP: Gross domestic product
LCU: Local currency unit
MVA: Medical vacuum aspiration
PAC: Post abortion care
PACCM: Post- abortion care costing methodology
PPP: Purchasing power parity
UE: Uterine evacuation
WHO: World health organization
