# Supplementary material for: The cost of post-abortion care (PAC): a systematic review
Source: BMC Health Serv Res. 2022 Mar 25;22:391. doi: 10.1186/s12913-022-07765-1 (PMC8953061; doi:10.1186/s12913-022-07765-1)
Supplement: Supplementary file 1 — Additional file 1. [file 12913_2022_7765_MOESM1_ESM.docx]

**Table 1. Search Terms**

**^Pubmed/Medline^**

|  | **Search terms** |
| --- | --- |
| Health system cost | (((((((((((((((((((health system cost*) OR (direct cost*)) OR (indirect cost*)) OR (cost analysis)) OR (cost–benefit analysis)) OR (cost-utility)) OR (cost-of-illness)) OR (health care cost*)) OR (“direct service costs” )) OR (“drug cost*”)) OR (“burden” )) OR (“hospital cost*”)) OR (“health expenditures” )) OR (“capital expenditures” )) OR (“direct medical cost*”)) OR (“supply cost*”)) OR (“personnel cost*”)) OR (“overhead cost*”)) OR (“capital cost*”)) OR (“economic*”)) |
|  | **AND** |
| **Post-abortion care** | ((((((((((((((("postabortion care") OR ("postabortion care"[MeSH Terms])) OR ("post abortion care"[MeSH Terms])) OR (postabortal[MeSH Terms])) OR ("post abortal"[MeSH Terms])) OR ("postabortion complication*"[MeSH Terms])) OR ("post abortion complication*"[MeSH Terms])) OR ("post-abortal complication*"[MeSH Terms])) OR ("incomplete abortion*"[MeSH Terms])) OR ("incomplete abortion"[MeSH Terms])) OR ("unsafe abortion*"[MeSH Terms])) OR ("unsafe abortion* complication*"[MeSH Terms]) |

**Cochrane**

#1 MeSH descriptor Abortion, Induced explode all trees with qualifier: AE

#2 MeSH descriptor Abortion, Induced explode all trees with qualifier: BL

#3 MeSH descriptor Abortion, Induced explode all trees with qualifier: CO

#4 MeSH descriptor Abortion, Induced explode all trees with qualifier: MO

#5 MeSH descriptor Abortion, Induced explode all trees with qualifier: ST

#6 MeSH descriptor Abortion, Criminal explode all trees with qualifier: AE

#7 MeSH descriptor Abortion, Criminal explode all trees with qualifier: CO

#8 MeSH descriptor Abortion, Criminal explode all trees with qualifier: MO

#9 MeSH descriptor Abortion, Legal explode all trees with qualifier: AE

#10 MeSH descriptor Abortion, Legal explode all trees with qualifier: MO

#11 MeSH descriptor Abortion, Septic explode all trees

#12 MeSH descriptor Abortion, Induced explode all trees

#13 MeSH descriptor Cost explode all trees

#14 MeSH descriptor Burden explode all trees

#15 MeSH descriptor Economic explode all trees

#16 MeSH descriptor Expenditure explode all trees

#17 MeSH descriptor Postabortioncare Care explode all trees

#18 MeSH descriptor economic explode all trees

#19 MeSH descriptor cost-benefit explode all trees

#20 (#1 OR #2 OR #3 OR #4 OR #5 OR #6 OR #7 OR #8 OR #9 OR #10 OR #11 OR #12)

#21 (#13 OR #14 OR #15 #16 OR #17 OR #18 OR #19 OR #20)

#22 (#21 AND #22)

**CINAHL**

S1 =(MH "Abortion+") or (MH "Induced Abortion") or (MH "Spontaneous Abortion") or (MH "Postabortion Care") or (MH "PAC") or (MH "Postabortion complications")

S2 =(MH "cost+") or (MH "burden") or (MH "expenditure+") or (MH "economic") or (MH "cost-benefit")

S3 = S1 and S2

**Psyinfo**

all (costs OR health cost* OR indirect cost* OR cost analysis OR cost–benefit analysis OR cost-utility OR cost-of-illness OR health care cost* OR “direct service costs” OR “drug cost*” OR “burden” OR “hospital cost*” OR “health expenditures” OR “capital expenditures” OR “direct medical cost*” OR “supply cost*” OR “personnel cost*” OR “overhead cost*” OR “capital cost*” OR “economic*” )

AND all "postabortion care" OR "postabortion care" OR "post abortion care" OR postabortal OR "post abortal" OR "postabortion complication*" OR "post abortion complication*" OR "post-abortal complication*" OR "incomplete abortion*" OR "incomplete abortion" OR "unsafe abortion*" OR "unsafe abortion* complication*"

^notes:^ search terms for cost related were developed based on Gordon, et a. 2015. ^c^search terms for abortion related were developed based on Tripney J, et al. 2011.
